# How to understand sports and traditional games and how to apply it to physical education. On the “Goal of Game”

**DOI:** 10.3389/fspor.2023.1123340

**Published:** 2023-02-28

**Authors:** J. P. Ribas, J. Hernández-Moreno, R. Díaz-Díaz, P. J. Borges-Hernández, J. V. Ruiz-Omeñaca, A. R. Jaqueira

**Affiliations:** ^1^ Teaching Faculty, Didactics of Languages, Arts and Sports, University of Málaga, Málaga, Spain; ^2^ Faculty of Physical Activity and Sport Sciences, Didactics of Corporal Expression, University of Las Palmas de Gran Canaria. Las Palmas de Gran Canaria, Gran Canaria, Spain; ^3^ Teaching Faculty, Specific Didactic, University of La Laguna. San Cristobal de La Laguna, Santa Cruz de Tenerife, Spain; ^4^ Teaching Faculty, Educational Sciences, University of La Rioja, La Rioja, Spain; ^5^ Faculty of Sport Sciences and Physical Education, Laboratório de Jogos, Recreação, Lutas Tradicionais e Capoeira, University of Coimbra, Coimbra, Portugal

**Keywords:** goal of game, traditional games, sports, TGfU, motor praxeology, physical education, motor-goal

## Abstract

**Introduction:**

Does philosopher's stone exist in physical education? It could be said that teaching games for understanding approach (TGfU) keeps turning everything it touches into gold: its presence in the educational centers, its volume of publications, the way of teaching games and sports, its connections with other approaches, its game categories, learning transferable principles of play. But… no, all that glitters is not gold. There are TGfU issues that should be improved. For example, these categories are disconnected from each other because TGfU lacks classification criteria. The “goal of game” is a concept that has been studied, but it has not been applied to physical education. The aim of the article is to show how to deepen the understanding sports and traditional games from the “goal of game”, and to propose its applicability to physical education.

**Methods:**

The traits of “goal of game” will be identified by investigating two close concepts, “prelusory goal” (formalist philosophy of sport) and “motor-goal” (motor praxeology).

**Results:**

The traits of “goal of game” concept: main-motor-problem, described in the game rules and that the players will try to solve during the game dynamics. The “goal of game” chances: (1) It allows us to understand sports and traditional games based on their internal logic (2) It allows us to classify traditional games and sports based on classification criteria and that can be useful to organize the physical education program; (3) It allows us to deepen the understanding of sporting games and their applicability to physical education: on the one hand, proposing progressively more specific goal of game options and, on the other hand, proposing a network model of intentions of play to understand the game dynamics and to design learning tasks.

**Conclusions:**

The conclusions collect some properties of the “goal of game” concept in order to propose its applicability in physical education students learning: identify and compare the main-motor-problems of the games; solve these problems during the game dynamics; transfer the procedures used to solve other games. The goal(d) of game amazes us; maybe physical education teachers are curious to continue discovering this wonderful treasure.

## Introduction

Teaching Games for Understanding approach (TGfU) is notable because some merits. Its progressive diffusion in physical education (PE) (1, 2) has led to a substantial bibliography for practical application, theoretical foundations, and research (3–7).

Some teaching and research approaches have come closer to TGfU, such us motor praxeology (8, 9), constraints-led approach (CLA) (10), game-centred approaches (GCA) (4, 11), game sense (12, 13).

TGfU has modified the way of teaching and learning games and sports, making the students to focus their attention to the logic of the game dynamics (14). Compared to the traditional skill drill technical model (15), TGfU achieves higher motivation (16), strategic knowledge transfer (17) and student's connection to the activity (18).

The summary is that “*The Teaching Games for Understanding (TGfU) approach for games teaching in physical education is one such increasingly popular teaching approach that advocates a learner-centered orientation, with emphasis on exploratory learning within “gamelike” situations* (2)” (p. 252). It could be said that TGfU is the philosopher's stone of PE: it turns everything it touches into gold.

Furthermore, TGfU proposes categories, where each category contains games and sports whose design logics and game dynamics logics are similar: “*If teachers select or sample different games from the same category, children can be led to understand similarities between apparently dissimilar games within a game form. For example, basketball and soccer, as invasion games, can explore common principles of attack and defense. Also, differences between apparently similar games can be compared, such as tennis and badminton as net games* (19)” (*p*. 30).

Time is tight in PE. The student will not have to learn and practice countless games and sports in PE if a curriculum is proposed from game and sports categories (20). The objective is for the student to understand and retain the structures and game principles of each category and to reuse them in the practice of sports and games with similar internal logics (transfer) (17, 21,22).

The initial TGfU grouped games and similar sports into four broad categories. They are games similar to each other because they coincide in their designs and in their (tactical) principles of play (23). For instance, invasion games” (soccer, basketball, hockey, rugby) share: a goal or similar target for scoring, invading territory to make space in attack and the containment of space in defense.

This means that during the game dynamics of one and the other games and sports that belong to the same category, the student performs similar procedures (20, 24): similar intentions of play, similar decision-making, similar interpretations of game situations. It is worth pointing out this way of acting in PE proposed by the TGfU. This is precisely what the development of this paper will focus on (see Table 1).

**Table 1:** The four categories of games chosen by the TGfU approach (this is a selection from the original table by Butler (25).

		Categories of games		
	Target	Striking/Fielding	Net/Wall	Invasion
Examples	Archery, bowls, golf, bowling, croquet, curling, pool	Baseball, cricket, kickback, softball rounders	Net: badminton, pickle-ball tennis table-tennis, volleyball	Basketball, field ice hockey, soccer, lacrosse, water polo, football, ultimate frisbee
			Wall: handball racquetball, squash	
Main intention of game	To send away an object and make contact with a specific, stationary target in fewer attempts than opponent	To place the ball away from fielders in order to run the bases and score more runs than the opponents	To send ball back to opponents so that they unable to return it or are forced to make an error	To invade the opponents’ defending area to score a goal while simultaneously protecting own goal

The diversity of learning experiences in PE ensures the improvement of the spheres of the person (26) and of the student's motor-behaviour (27): affective, cognitive, motor and relational. But if a student spends many hours learning and practicing the same type of very similar games he will have less opportunity to take time to experience other beneficial experiences. For example, learning more and more invasion games (soccer, basketball, hockey, tag rugby, ultimate, lacrosse) implies a repetition of similar game experiences. Then, “*The focus of TGfU is to design learning experiences for individuals to acquire tactical skills of the major games through playing modified versions of target games considered suitable for their current physical, intellectual, and social states of development* (2)” (*p*. 253).

The learning and practice of games of diversity of categories ensures the diversity of experiences and its consequent influence in the spheres of the person (28, 29). This has implied that more and more categories have been added to the TGfU model over the years (6), for example, wrestling games (judo, wrestling, canarian wrestling) and combative games (taekwondo, fencing, canary stick game) (30), traditional sports and games whose goal of game is for players to throw a moving-object at other players' bodies (ball-tag games, paintball, dodgeball, sitting-ball) (31) and tag games (cops and robbers, kabbadi, kho-kho) (32, 33).

The games and sports included in these other categories also meet the requirements of being games similar to each other because they coincide in their designs and in their structures and (tactical) principles of play (23, 25). Then, the student performs similar procedures in these sports and traditional games during the game dynamics (20, 24). Then, they can meet the requirement of transfer between games belonging to a category (17, 22). And, finally, they can meet the other requirements of being major games, target games and being games considered suitable for their current physical, intellectual, and social states of development (2).

Other kinds of sports and traditional games that can perfectly meet part or all of the above requirements of the TGfU: traditional sports and games whose goals is for players to reach a goal-place (relay races, puss in the corner, musical chairs, king of the mountain, green light-red light), acrobatic games (artistic gymnastics, capoeira, trampoline), juggling, games of building human towers (castellers, acro-sport), games with music and rhythm (jump rope, rhythmic gymnastics, clapping hand games, aerobics). Definitively, more games and sports categories are needed in PE (29).

A PE curriculum that plans the practice of sports and games based on categories is more advisable than a curriculum that chooses a list of countless sports and games with no connection to one another In line with the proposals of the TGfU (23), it is advisable to distribute the wide universe of games and sports by exhaustively grouping them into a finite number of large categories (34).

To organize a homogeneous and exhaustive system of game and sports categories, it is not enough to identify the traits that differentiate some categories from others (20) (specific differences). It is also essential to identify what all the categories have in common with each other (near gender), that is, classification criteria are needed. But TGfU lacks classification criteria to organize and connect its categories. All that glitters in TGfU may not be gold.

In this sense, the classification criteria belonging to internal logic are appropriate in PE because they allow us to understand games and sports internal structures (34). Elements of internal logic are: goal of game, game space, game time, motor-communication and materials (34). Motor praxeology has developed categories using classification criteria the “type of space” and the “type of motor-communication” (35), but has not proposed categories using “type of goal of game” as classification criterion.

The “goal of game” is significant: it has been insistently covered since the 1960s (36), until the present (37). However, the goal of game has not been used in PE to understand games and sports and for its applicability in student learning. The “goal of game” is a treasure to discover in PE.

The aim of the article is to show how to deepen the understanding sports and traditional games from the “goal of game”, and to propose its applicability to physical education.

## “*Goal of game*” and understanding of sports and traditional games

In the 1960s, the debate on the pre-lusory goal was sparked among formalists, a current within the philosophy of sport that tries to describe and define the concepts “game” and “sport” from the rules (38, 39). The triggering article published in 1967 “What Is a Game?” is signed by Bernard Suits. The debate continues in the present.

Since the 1990s, and after the publication in 1981 of the book “Contribution à un lexique commenté en science de l'action mortice” (40), motor praxeology (science of motor action) has developed concepts, classifications, some research and some proposals of applicability in PE, based on the “motor-goal” (37).

 We will focus on the contributions of the formalists and motor praxeology to understand the traits of the “goal of game” concept.

*The “goal of game” is an internal component of the game*. Suits (36) uses the term “lusory goal” (“lusory”, from ludus, “game” in Latin) that means “the goal of game” and refers to a state of affairs to be reached during the game (reaching the goal of game). Suits (41) renamed it “pre-lusory goal” and both terms have come to be used with similar meanings (42).

However (43), describes the differences between the two states of affairs to be achieved. It is the same argument defended by Devine and Lopez-Frias (39): “*Games are goal-directed activities. Each game has two distinct goals: a “lusory” goal and a “prelusory” goal. The pre-lusory goal is a specific state of affairs that players are trying to achieve: putting the ball in the hole in golf, crossing the bar in the high jump, and crossing the line in the marathon. These goals can be achieved prior to the formation of a game. For example, I can put a golf ball in a hole even though no golf game has started, or I can jump over a bar even though no high jump competition is in progress. The lusory goal is to win. This can only be achieved in the context of organized play*.” (digital version).

For motor praxeology, the motor-goal is an element belonging to the internal logic of games and sports (44). Parlebas (34) understands internal logic as the logic of game design (sports rules, traditional game rules) and as the logic of game dynamics (principles of play, decision-making, tactical problems, intentions of play, perceptions, interpretations of the situation of play).

*The “goal of game” is the problem to be solved by the players*. Torres (45) uses the term “playful problems of the game”. For Schell (46) (*p*. 37) “*A game is a problem-solving activity, approached with a playful attitude*”. According to Kretchmar (47) (*p*. 12) “*To play a game, we look for (or, as far as necessary, invent) a good problem just so that we can encounter it and try to solve it*”. And according to motor praxeology, motor-goal is the demand to be achieved by the participants (44).

*The “goal of game”-problems are motor-goals*. Parlebas (34) refers to the “motor-task” of sporting games (traditional games, sports), and Lagardera and Lavega (28) say that physical activities and sports are “… *motor-oriented situations, carried out by means of our motor faculties, and with a strictly motor purpose: to score a goal, to pass the bar, to exercise*” (*p*. 50) (respectively in soccer, pole vaulting, and cycling). Rodríguez-Ribas (48) proposed the term “motor-goal” after concluding in his inductive study that all physical activities and sports coincide in that “… *the goal to be achieved is of a motor nature*” (*p*. 31).

Formalists as Suits (49) (*p*. 2) says that sports are “… *competitive events involving a variety of physical human skills (usually in combination with others), in which the superior participant is judged to have exhibited those skills in a superior manner*”. And Meier (50) (*p*. 24) says that “… *a game may also properly be called a sport if it possesses the additional characteristic of requiring participants to demonstrate physical dexterity or skill in the achievement of its objective*”.

The motor-goal is not only a feature common to all games and sports, it is also a criterion that differentiates (discriminates) what is a motor-game or sport from other games (48, 51, 52). Non-motor games (board games, chess, e-sports) have no motor-problems to solve.

And finally, *the “goal of game”-problems are the main problems* that players solve during the game dynamics (44).

**Figure 1:**
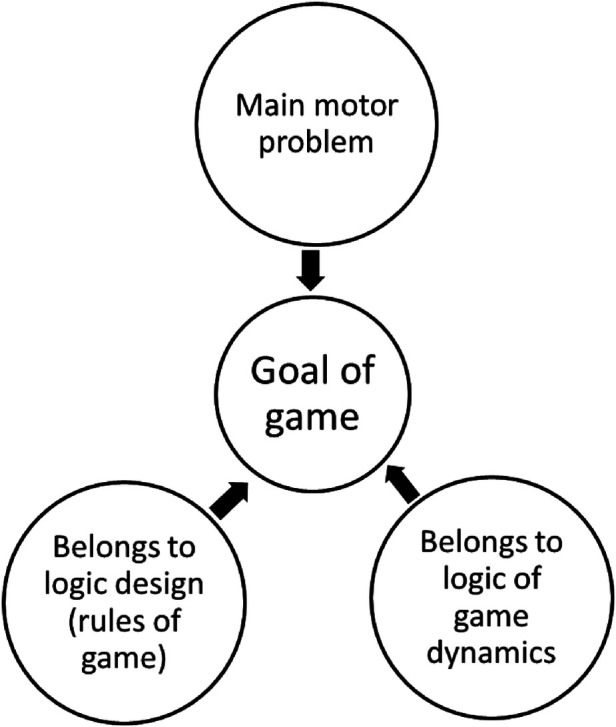
Traits that identify the “goal of game” of sports, traditional games and modified games.

Summarizing, in traditional games, sports and modified or invented games, the concept “goal of game” refers to a main-motor-problem, described in the rules of the game and that players will try to solve during the game dynamics (see Figure 1).

Let's check with an example if there is concordance between the formulation of the concept and reality. The two basketball goals of game are: “*The aim of each team is to score in the opponents’ basket and to prevent the other team from scoring* (53)” (art 1.1, *p*. 6). Both are the two main problems that players must solve (article 1.1). Both are motor-problems (getting the ball into the opponents' basket, and preventing them from doing so). Both problems are described in the rules (page 6 and later). Players try to achieve these two goals of game during the game dynamics (playing a basketball game).

The goal of game facilitates the understanding of the internal logic of the game. A short sentence summarizes what the game consists of and summarizes what elements are necessary for the game (in basketball: one ball, two baskets, two opposing teams).

Other examples of goals of game but in traditional games are:

- Dodgeball (two goals of game): hit the ball into the opponents' body, and prevent the opponents from doing so.
- Green light-red light (two goals of game): get to the goal line but avoid moving when “it” turns around, and restart from the starting line if “it” sees you move when it turns around.
- Puss in the corner (two goals of game): to reach a free corner before another player, and to prevent or encourage other players to reach a corner.

Is it possible to use the “goal of game” as a classification criterion for sports and traditional games? The elaboration of a specific classification in PE based on the “goal of game” criterion requires answering the question: “What types of goals of game exist in sports and traditional games?” And specifically, which categories of *main-motor-problems* can be found in the rules of sports and traditional games.

Parlebas (35) states that the goals of game of traditional games and sports included in the so-called “sporting games” are of the space type. “*The spatial goals are the poles around which the acts of the game gravitate*” (*p*. 181). Examples of sporting games are invasion games, net/wall games, tag games, fighting games and running games. The goals of game of the sporting games are synthesized in “to overcome motor-spaces”.

Also from motor praxeology, Mateu and Bortoleto (54) (*p*. 133) propose “motor-forms”, where “… *the purpose of the motor action: oriented by the production of meaning and by the morphokinetic character*” determine the goals of game of expressive traditional games and aesthetic sports. Examples of this type of sports and traditional games are acrobatic games, juggling games, games of building human towers, games with thematic meanings, games with music and rhythm. The goals of game of motor-expression games are summarized in “to obtain motor-forms”.

Formalist sports philosophers (42, 55, 56) distinguish the two large categories of games as well. Kretchmar (57) clarifies Suits (48) by adding that what he calls “performances” is guided by aesthetic factors.

Different goals of game suggest new definitions. Sporting games: “Sports and traditional games whose goals of game imply to overcome motor-spaces”. Motor-expression games: “Sports and traditional games whose goals of game imply to obtain motor-forms”.

The goal of game allows the understanding of games and sports. The two large categories distribute sports and traditional games of the PE curriculum based on two different goals of game (see Table 2).

**Table 2:** The two large categories of sports and traditional games according to their goals of game, and some examples.

	**Categories**	
	Sporting Games	Motor-Expression Games
	**Goals of game**	
	*“to overcome motor-spaces”*	*“to obtain motor-forms”*
	**Examples**	
Traditional Games	Traditional sporting games: Puss in the corner, blob tag, capture the flag, ten passes, sitting ball	Expressive traditional games: Castellers, diabolo, jump rope, the mirror, jogo (capoeira), yo-yo
Sports	Basketball, soccer, baseball, kabaddi, golf, tennis, bowling, kumite (karate) dodgeball, volleyball, ultimate	Aesthetics sports: Surfing, figure skating, dance sport, dunk contest, artistic gymnastics

## Deepen the understanding of sports and traditional games. The sporting games in PE

We dedicate this section to deepen the understanding of sports and traditional games. We'll use “goal of game” to apply specifically to sporting games. Deepening the understanding of sports and traditional games that are sporting games using their goals of game requires answering the question: What are the different options “to overcome motor-spaces” in sports and traditional games?

According to the formalist Kretchmar (47) (*p*. 6), the goal game problems include two constituents: “… *gamewriting is a process of manipulating means and ends for purposes of producing ‘just right’ problems. Frequently, it is a combination of both. Naismith's invention of basketball is a case in point. He problematized the prelusory goal of ‘ball-through-basket’ by elevating the basket. He also limited permissible means for achieving this state of affairs by prohibiting the use of ladders and by allowing interference by defenders. The combination of the two produced a provocative game problem*”.

Adding means and conditions to “to overcome motor-spaces” produces new, more concrete problems, i.e., more concrete goals of game. From the motor praxeology, Parlebas (35) (*p*. 177) differentiates in sporting games between space as “*distance to travel”* and space as “*target to achieve”.* The distance to travel *“It is the distance a ball or puck is made to travel; It is also, and above all, the distance to travel oneself*”. Regarding the target to be achieved, he distinguishes between “*The material targets: almost always fixed, they correspond to conditioned places*” (*p*. 181) and human targets, in which “*The space to be achieved is a dynamic human space…*” (*p*. 182); it is a human target. According to the pointed out possibilities, two components are distinguished for the goals of game “to overcome motor-spaces”:

- The arrival component has two options: 1. A target or a goal; 2. Players are the (dynamic) target to achieve.
- The component that will overcome the motor-space to the arrival component has two options: 1.The players themselves will overcome the motor-space to the arrival component; 2. The moving-objects (ball, disc, dart…) used by the players are those that will overcome the motor-space to the arrival component.

**Figure 2:**
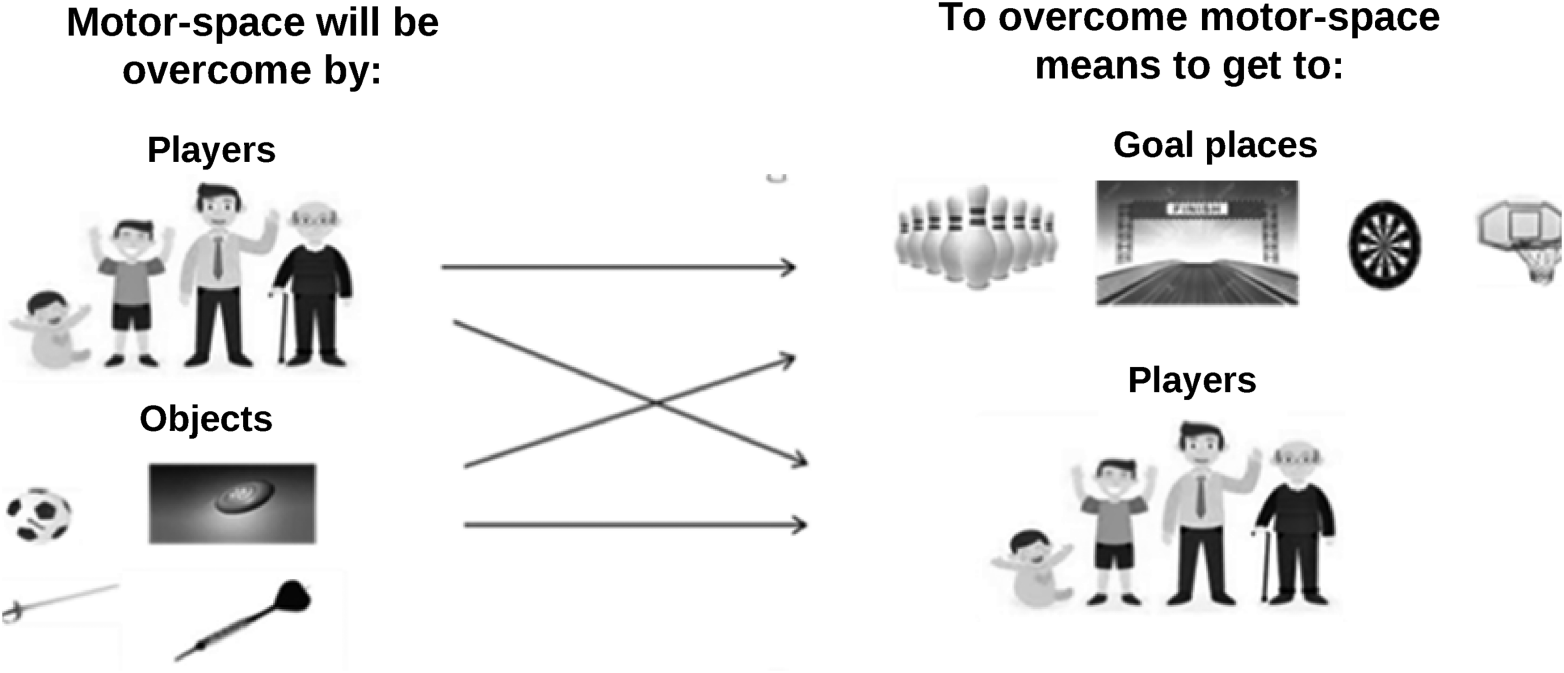
Combinations between the two options of the two components based on the goal of game “to overcome motor-spaces” (arrival component and component that overcomes the motor-space to the arrival component).

Let's act deductively. The combination between the two options of both component (see Figure 2) implies a classification of four more specific types of the goal of game “to overcome motor-spaces” (which will be named number 1):

1.1. To overcome motor-spaces (players themselves) to the goal.
1.2. To overcome interpersonal motor-spaces.
1.3. To overcome motor-spaces (moving-objects) to the goal or target.
1.4. To overcome motor-spaces (moving-objects) to players.

Which sports or traditional games contain one or more of these goals of game? For example, basketball and all invasion games contain the goal of game. 1.3. [“To overcome motor-spaces (moving-objects) to the goal or target”]; blob tag and all tag games contain the 1.2. goal of game; baseball and all striking/fielding games contain the goals of game 1.1, 1.3. and 1.4.; paintball and dodgeball contain 1.4.

For further deepening the understanding of traditional games and sports, it will be necessary to deduce their goals of game with a higher concreteness degree. For this purpose, more conditions will be added to the four goals of game. Here are some condition options that can be added:

- The arrival component can be static or be dynamic. For example, the basket in basketball is static, while the human paintball target or the skeet shooting target are in motion.
- The arrival component is usually delimited and localized (e.g., goal, target, bowling, other participants), but there are also variable spaces as a goal, for example, in long jumping or throwing objects away (throwing in athletics).
- Participants may cooperate to get moving-objects or other participants to overcome the motor-space; on the other hand, there are sports and traditional games in which opponents will prevent it.

The following classification of game goals has three concreteness degrees. Some examples of traditional games and sports that contain one or more game goals have been included.

1. “To overcome motor-spaces”
  1.1. To overcome motor-spaces (players themselves) to the goal:
  1.1.1.1. Perform races/runs (and/or preventing the opponent from doing so). E.g. relays, parkour, striking games (baseball, cricket, rounders), climbing.
  1.1.2. Perform height jumping or distance or obstacles jumping (long jump, pole vaulting, ski jumping, bungee jumping).
  1.1.3. Occupy spaces (and/or preventing the opponent from doing so) (puss in the corner, musical chairs, king of the mountain).
  1.2. To overcome interpersonal motor-spaces:
    1.2.1. Hit/touch others and preventing the opponent from doing so. E.g., combative sports (boxing, fencing, taekwondo), tag games (cops and robbers).
    1.2.2. Immobilize others and preventing the opponent from doing so (judo, wrestling).
    1.2.3. Knock down/excluding others from a space and preventing the opponent from doing so. E.g. judo, sumo wrestling, fencing, traditional wrestling (canarian wrestling).
    1.2.4. Group with others (and/or preventing the opponent from doing so). E.g. running to group, Romeo and Juliet.
    1.3. To overcome motor-spaces (moving-objects) to the goal or target (examples of moving-objects: ball, disc, puck, javelin):
    1.3.1. Put the moving-object to a target or goal and to prevent the opponents from doing so. E.g. invasion games (soccer, basketball), conquest of the flag, kinball, tchoukball, striking games.
    1.3.2. Prevent the opponent from forwarding the moving-object to a target or goal. E.g. net/wall games (volley games, tennis games), spikeball.
    1.3.3. Throw, shooting or hitting moving-object accurately towards a target. E.g. target games (golf, billiards, bocce, bowling, three-point contest, croquet).
    1.3.4. Throw, hitting moving-object at a distance (athletics throws, striking games).
    1.3.5. Lifting or dragging objects. (weight lifting).
  1.4. To overcome motor-spaces (moving-objects) to players:
    1.4.1. Throwing or passing a moving-object to others (and/or preventing the opponent from doing so) E.g., dodgeball, sitting-ball, paintball, ten passes, rondo, striking games, throwing the fresbee to others.
    1.4.2. Making a moving-object return (auto-passes, bouncing the ball, boomerang).

This is a classification of goals of game. For example, striking/fielding games contain four different goals of game and, therefore, appear in several categories of goals of game (1.1.1. Performing races/runs; 1.3.1. Get the moving-object to a target; 1.3.4. Throw, hitting moving-object at a distance; 1.4.1 Throw, pass a moving-object to others.

This classification of goals of game has three concreteness degrees, but further concreteness of goal of game can be made to expand the understanding of similar games (or groups of games). For example, starting from the goal of game of invasion sports 1.3.1. “Put the moving-object to a goal and prevent the opponents from doing so”, two more concreteness degrees can be specified:

Concreteness level 4 (1.3.1.1): “To put the moving-object into the opponents’ goal and to prevent them from doing so”, of invasion sports with goal (soccer, field hockey, polo, handball).

Concreteness grade 5 (1.3.1.1.1.1): “To put the ball into the opponents’ goal and preventing them from doing so, directing the ball with any part of the body except arms and hands”, from soccer [indoor soccer (5 players), soccer-7, soccer-11, jorkyball, beach-soccer].

To correctly express a goal of game requires the infinitive of a verb with the meaning of a problem to be solved, and described with the means contained in the problem (Kretchmar, 2019). For example, the basketball goals of game (two baskets) would be correctly expressed as follows: “to put the ball into the opponents’ basket, and to prevent the opponents from putting the ball into our basket”.

The goals of game allow a deeper understanding of sporting games from their internal logic (34), that is, from the logic of design (the rules of the game) and from the logic of game dynamics. We have deepened the logic of design of sports and traditional games through goals of game. Is it possible to deepen the understanding of the logic of the game dynamics of sports and traditional games through the goals of game?

As formalist sports philosophers point out, playing a game is to attempt to achieve the goals of game (47, 58, 59). In the same sense, Bayer (59) (*p*. 62) specifies for invasion games that: “… *each player will carry out his action on the playing field, with an intention (and the meaning that is attached to it) that will modify the present situation and it will motivate on the part of the other players (in order to preserve the balance of the system) some intentions that will be articulated among themselves*”.

**Figure 3:**
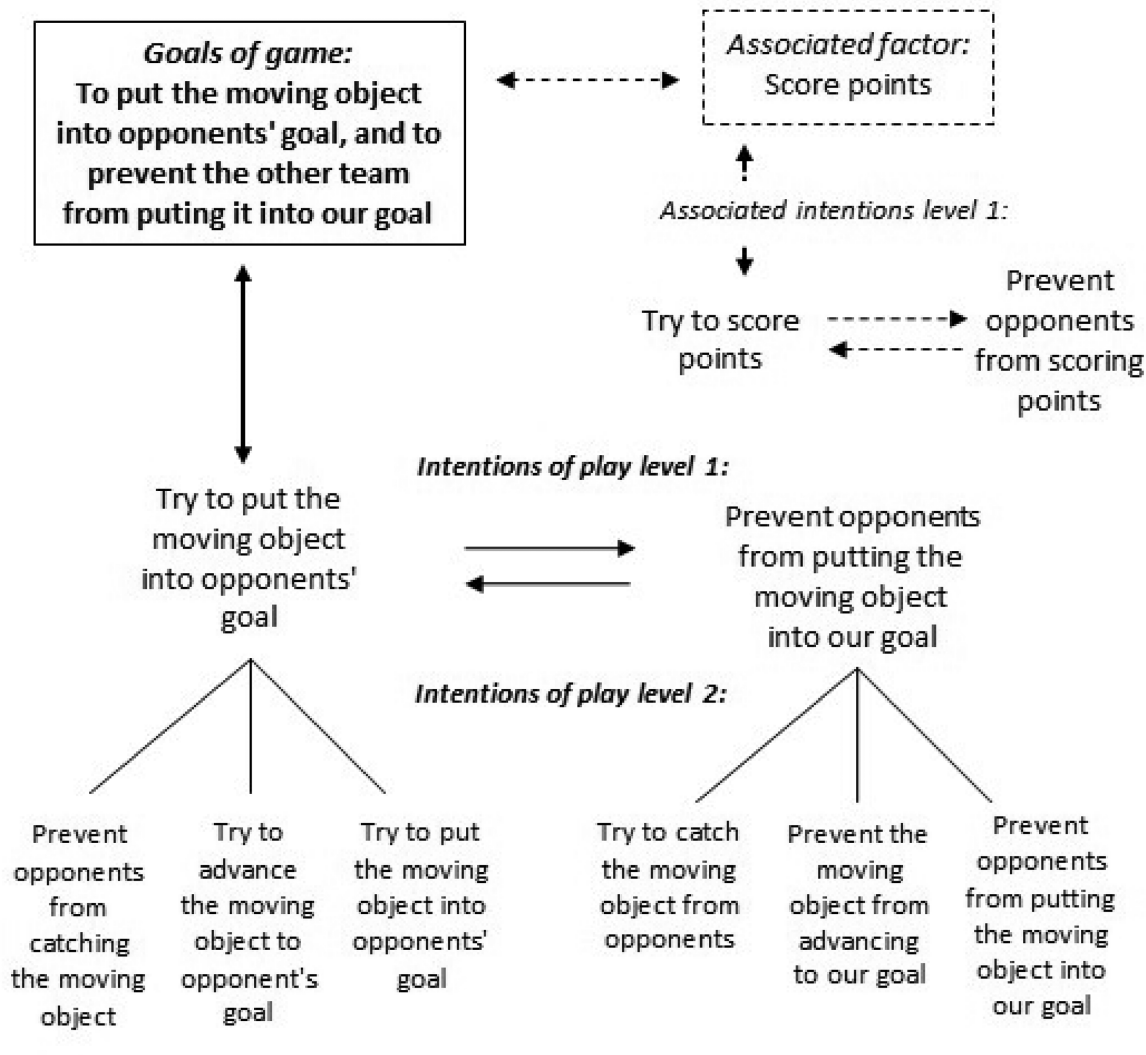
Network model of intentions of play of invasion games (according to the principles of play proposed by Bayer (60).

Curiously, the principles of play of invasion games in Bayer (60) (from phenomenology) are the same ones used by Bell and Hopper (61) (from TGfU) to design invasion game learning tasks. We propose a “network of intentions of play” that collects the articulation of levels of intentions of play of the players during the game dynamics, and that are triggered from the goals of game. The learning tasks are associated to each intention of play. The different levels of intentions of play (level 1, level 2, level 3…) allow us to create tasks for different levels of learning (see Figure 3).

Regarding PE, the similarities between the intentions of play of two games suggest coincidental teachings and positive transfers between those games; and the notable differences between their intentions of play suggest a differentiated teaching of those games. And going back to what was stated in the introduction of the article, the transfer of procedures to solve different games is one of the TGfU's working hypotheses (17, 21, 22)^,^.

## Conclusions

“*Goal of game*” refers to a main-motor-problem, described in the rules of the game of sports and traditional games, and that the players will try to solve during game dynamics. For example, the two goals of game of dodgeball are to hit the ball into the opponents' body, and prevent the opponents from doing so; and the two goals of game of basketball are to put the ball into the opponents' basket, and to prevent the opponents from putting the ball into our basket.

Regarding the questions in the article title “how to understand traditional games and sports and how to apply it to physical education”, we've have compiled some of the “goal of game” properties taken from the sections of this article, and we propose applicability options in PE for student learning:

- The goal of game is described with a brief phrase, which summarizes what the game consists of and summarizes what elements are necessary for the game. Students can identify the main-motor-problems that they will have to solve in each game (or group of similar games).
- The goal of game is a motor-problem. Students can discriminate between motor games (traditional games and sports) compared to non-motor games (board games, chess, e-sports). Non-motor games have no-motor problems to solve.
- The goal of game serves to classify. Students can compare games by recognizing similarities or differences between main-motor-problems from different games (or a group of games).
- The game dynamics is deduced from the goals of game. Students can solve each main-motor-problem by selecting intentions of play.
- The intentions of play could be transferable. Students can perform similar procedures to solve a main-motor-problem (goal of game) that belongs to two different games (or group of games).

“Goals of game” and “intentions of play” can help PE teachers to plan the program of PE, to design teaching units and sessions, to design modified games and to control the monitoring of student learning.

The “goal of game” is a concept that allows deepening the understanding of sports and traditional games, but it had not been applied to PE before. The authors are aware that what is provided in this article is a first approximation: the goal(d) of game amazes us; maybe PE teachers are curious to continue discovering this wonderful treasure. The “goal of game” can enrich the foundations, research and its applicability from motor praxeology, from TGfU and from the philosophy sport.

## Data Availability

The original contributions presented in the study are included in the article/Supplementary Material, further inquiries can be directed to the corresponding author/s.
